# Decoupled Multiplicative Physics-Guided Neural Network for Extrapolative Modeling of GaN HEMT

**DOI:** 10.3390/mi17101181

**Published:** 2026-10-12

**Authors:** Yongchuan Tang, Longxiang Hou, He Guan, Borui Deng, Haolei Zhu, Ying Wang

**Affiliations:** 1 School of Integrated Circuits and Microelectronics, Northwestern Polytechnical University, Xi’an 710072, China; houlongxiang@mail.nwpu.edu.cn (L.H.); dengborui@mail.nwpu.edu.cn (B.D.); zhuhl@mail.nwpu.edu.cn (H.Z.);; 2 Research & Development Institute of Northwestern Polytechnical University in Shenzhen, Shenzhen 518057, China

**Keywords:** GaN HEMT, physics-guided neural network, DC self-heating modeling, extrapolative modeling, multiplicative residual

## Abstract

Rapid direct-current (DC) surrogate modeling of gallium nitride (GaN) high-electron-mobility transistors (HEMTs) requires accurate prediction of the current-capability, conduction-loss, and self-heating indicators used in geometry screening. This work presents an electrothermal decoupled multiplicative physics-guided neural network (ET-DM-PGNN) for this task. It combines a gray-box backbone, separate high- and low-field polarization channels, a latent DC self-heating update, and bounded target-specific multiplicative residuals. A two-stage strategy first calibrates the complete surrogate and then refines the current branch while preserving resistance and temperature. On 32 interstitial samples, ET-DM-PGNN achieves $R^{2}>0.997$, with mean absolute percentage errors of 0.13%, 0.15%, and 0.04% for $I_{\max}$, $R_{\mathrm{on}}$, and $T_{\max}$, respectively. The corresponding errors on nine external cases are 1.46%, 0.94%, and 0.74%. A leakage-controlled, three-seed level-held-out audit yields errors of 0.44–6.40% in four composition and lateral-geometry directions, whereas holding out the 0.015 µm barrier level produces errors of 12.41–21.21% under the tested training protocol. Across 365 geometries, 4 updates differ from a 12-step reference by at most 0.0076%. Numerically validated at $V_{\mathrm{gs}}=3$ V using TCAD DC sweeps extending to $V_{\mathrm{ds}}=15$ V, the surrogate supports rapid fixed-protocol geometry screening and identifies the ultrathin-barrier direction as the least reliable tested extrapolation regime.

## 1. Introduction

Gallium nitride high-electron-mobility transistors (GaN HEMTs) are important in radio-frequency (RF) and power-electronics applications because of their wide bandgap, high critical breakdown electric field, and high electron mobility [1,2,3]. GaN-on-Si substrates and CMOS-compatible fabrication have also supported lower-cost, larger-scale integration [4,5]. Consequently, accurate modeling of direct-current (DC) characteristics—including output characteristics, transfer characteristics, threshold voltage, and on-state resistance—is important for device optimization, circuit design, and reliability analysis [6,7,8,9,10,11]. During early geometry exploration, maximum current, on-state resistance, and maximum lattice temperature provide a compact screening vector for current capability, conduction loss, and steady-state self-heating, respectively.

Each conventional modeling route has practical limitations. Although technology computer-aided design (TCAD) provides detailed physics-based numerical simulations, repeated coupled DC electrothermal sweeps can incur substantial computational cost and convergence difficulties [12,13,14,15]. Physics-based compact models, represented by ASM-HEMT [16] and MVSG [17], are widely used in industry, but their nonlinear parameter-extraction procedures frequently demand substantial human effort.

Recent learning-assisted device models increasingly combine neural approximation with physical knowledge. Physics-informed neural networks (PINNs) incorporate governing equations and boundary conditions through residual-based objectives [18,19]. In device-level surrogate modeling, physics-guided neural networks (PGNNs) and hybrid compact-model/neural-network approaches instead place reduced device relations in the forward architecture and learn their remaining discrepancy [20,21,22,23,24]. GaN HEMT surrogates remain sensitive to multi-output optimization and extrapolation beyond the sampled grid [25], while recent SiC MOSFET work extends physics-informed modeling to static and switching characteristics [26]. For geometry screening, a useful multidimensional surrogate should retain the main geometry, polarization, and steady-state self-heating trends, keep its outputs feasible, and be tested explicitly outside the training grid.

We address this problem with an electrothermal decoupled multiplicative physics-guided neural network (ET-DM-PGNN). It predicts $I_{\max}$, $R_{\mathrm{on}}$, and $T_{\max}$ to rank candidate geometries before selected designs are confirmed by full TCAD simulation. The architecture combines separate high- and low-field polarization mappings, a latent DC self-heating update, bounded multiplicative residuals, and branch-selective training. Evaluation uses independent interstitial samples, external cases, and leakage-controlled level-held-out splits along all five structural coordinates. The contribution lies in this GaN-specific architecture and its evaluation under three complementary extrapolation protocols.

The remainder of this paper is organized as follows. Section 2 presents the physical representation and training process of the proposed model. Section 3 examines training convergence, numerical closure, interpolation and extrapolation accuracy, directional level-held-out performance, and baseline comparisons. Section 4 concludes this paper.

## 2. Electrothermal Decoupled Multiplicative Physics-Guided Architecture

Purely data-driven surrogates may fit sampled characteristics yet produce electrically or thermally infeasible responses outside the sampled grid. In this work, “physics-guided” denotes the use of low-order geometry, polarization, transport-length, and steady-state power–temperature relations before neural branches learn bounded discrepancies. Because no governing-equation residual is used, the architecture is classified as a PGNN. Decoupling is implemented through separate low- and high-field polarization pathways and target-specific residual branches, while the latent DC self-heating update retains cross-target linkage. For $X=[x_{\mathrm{comp}},L_{g},L_{\mathrm{gs}},L_{\mathrm{gd}},t_{\mathrm{AlGaN}}]$, the model evaluates cold-state electrical bases, applies bounded residual corrections, performs the latent update, and maps the outputs to their feasible domains.

### 2.1. TCAD Simulations

**Figure 1:**
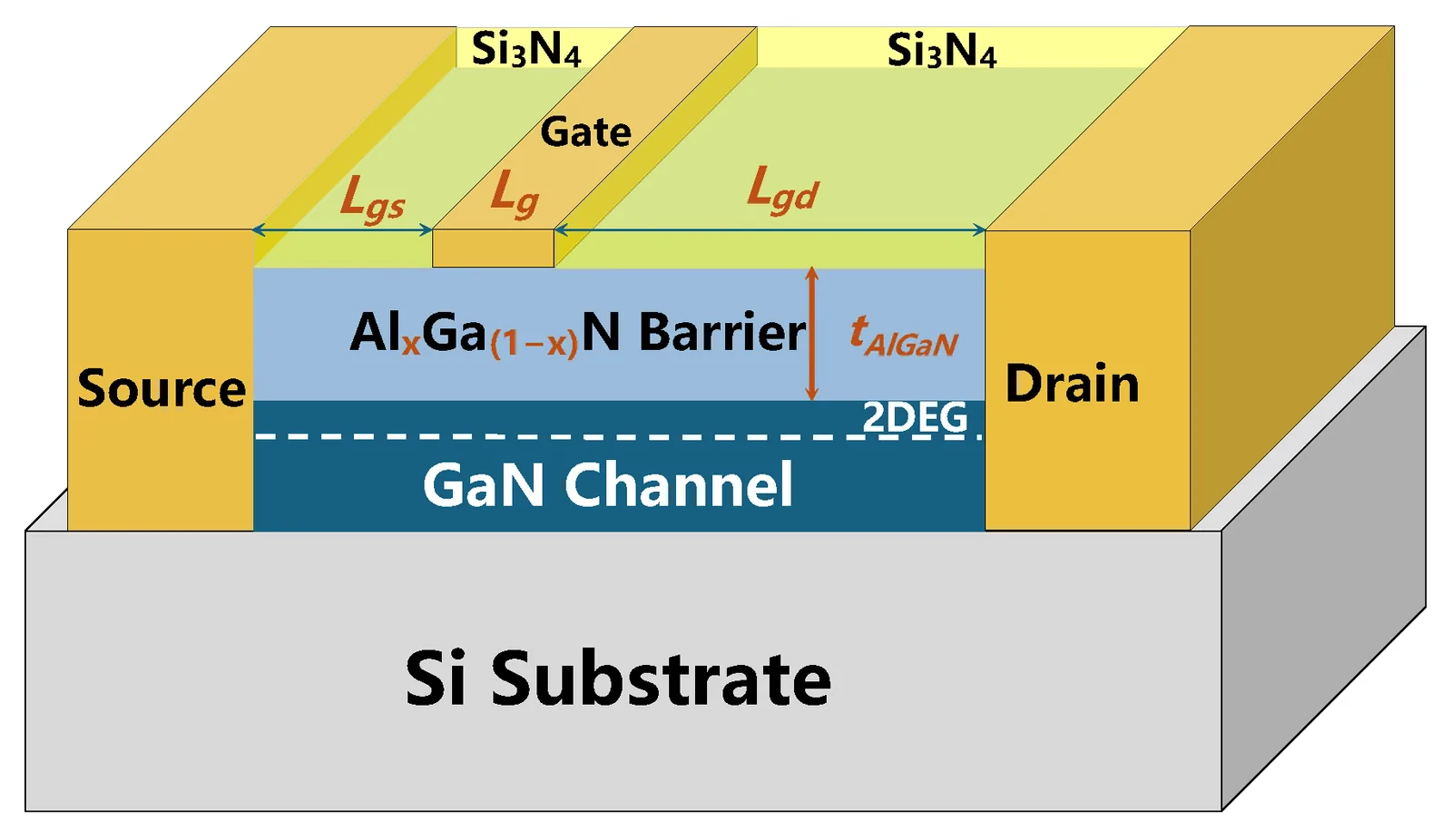
Schematic diagram of the simulated device structure.

The dataset was generated with Silvaco ATLAS from the cap-layer-free, depletion-mode Schottky-gate HEMT shown in Figure 1. This study considers its steady-state DC behavior and does not model transient trapping or virtual-gate effects. At $V_{\mathrm{gs}}=3$ V, the drain bias is swept from 0 to 15 V in 0.2 V increments. Three quantities are extracted from each sweep: $I_{\max}$ is the maximum drain current and represents current capability; $R_{\mathrm{on}}=V_{\mathrm{ds}}/I_{d}$ is evaluated at 0.4 V, the sampled point immediately below 0.5 V, and represents conduction loss in Ω·mm; and $T_{\max}$ is the maximum lattice temperature at $V_{\mathrm{ds}}=15$ V and represents steady-state self-heating severity. The targets summarize different regions of the same DC sweep rather than a single common bias point. A full-factorial design of 324 samples was constructed from five structural parameters: Al composition $x_{\mathrm{comp}}$, barrier thickness $t_{\mathrm{AlGaN}}$, gate-to-source spacing $L_{\mathrm{gs}}$, gate-to-drain spacing $L_{\mathrm{gd}}$, and gate length $L_{g}$. Table 1 lists the sampled structural levels.

The two-dimensional ATLAS deck contains an AlGaN barrier, GaN channel and buffer, and Si_3_N_4_ access-region passivation. The Si support in Figure 1 is represented thermally by the bottom boundary condition rather than as an explicitly meshed electrical region. Bandgap narrowing, field-dependent mobility, Shockley–Read–Hall recombination, the Albrecht electron-mobility model, and strain-dependent polarization with a polarization scale of 0.8 are enabled. Self-heating is solved with the lattice-temperature model at 300 K. Constant thermal conductivities of 0.88 and 1.5 W cm^−1^ K^−1^ are assigned to the AlGaN and GaN regions, respectively; the corresponding density/specific-heat pairs are 5.6 g cm^−3^/450 J kg^−1^ K^−1^ and 6.07 g cm^−3^/490 J kg^−1^ K^−1^. A 300 K thermal contact is imposed at the bottom of the 2 µm GaN buffer. The gate work function is 5.0 eV and the source/drain work functions are 3.93 eV. Newton iteration is used for the initial electrical solution and a block-Newton scheme is used for electrothermal sweeps, with a maximum of 20 trap iterations.

The nominal device width is 100 µm. The lateral grid is refined to 0.02 µm at the source-access, gate, and drain-access transitions, while the vertical grid uses 0.001 µm spacing across the passivation/barrier interfaces and is gradually relaxed in the buffer. These settings and the accompanying parameterized ATLAS deck provide a reproducible numerical reference across the five-dimensional geometry space. Published measurements and experimentally calibrated simulations support the qualitative links among dissipated power, self-heating, current reduction, and resistance increase in AlGaN/GaN structures [27,28]. Transfer to a fabrication process requires process-calibrated TCAD or measured data followed by retraining.

### 2.2. Physics-Guided Model Formulation

The gray-box backbone combines established device-level trends rather than reproducing a single compact model. Its current scale increases with polarization-induced channel charge and decreases with effective transport length; resistance increases with lateral length and decreases with available channel charge; and the steady-state temperature rise follows $\Delta T=PR_{\mathrm{th}}$ [16,17,29,30]. Equations (1)–(15) are trainable low-order realizations of these trends. The coefficients ${\bar{k}}_{1}$–${\bar{k}}_{5}$ calibrate electrical and geometrical scaling, $k_{6}$, ${\bar{k}}_{7}$, and ${\bar{k}}_{8}$ define the polarization proxy and its target-specific mappings, ${\bar{k}}_{9}$ sets the effective DC thermal scale, and ${\bar{k}}_{10}$ sets the electrical sensitivity to the predicted temperature rise. The backbone supplies interpretable trends and output scales, while bounded neural branches correct the remaining model-form discrepancy.

These coefficients are effective parameters of the calibrated device family rather than uniquely identified intrinsic material constants. Physical-unit inputs are supplied directly to the gray-box backbone, whereas normalized inputs are supplied to the residual networks.

#### 2.2.1. Cold-State Electrical Backbone

Neglecting self-heating, the cold-state current scale and on-state resistance are represented by

(1) $$I_{\mathrm{cold}}=\frac{{\bar{k}}_{1}P_{I_{\max}}}{L_{g}+{\bar{k}}_{5}L_{\mathrm{gs}}+{\bar{k}}_{2}}$$

(2) $$R_{\mathrm{cold}}={\bar{k}}_{3}\frac{L_{\mathrm{total}}}{P_{R_{\mathrm{on}}}+\varepsilon_{R}}+{\bar{k}}_{4}$$

(3) $$L_{\mathrm{total}}=L_{g}+L_{\mathrm{gs}}+L_{\mathrm{gd}}$$

where $L_{\mathrm{total}}$ is the total lateral length and $\varepsilon_{R}=10^{-3}$ is a dimensionless denominator floor. The coefficients ${\bar{k}}_{1}$–${\bar{k}}_{5}$ parameterize current scaling, channel and access-length modulation, resistance scaling, parasitic resistance, and source-side access feedback. Except for the separately bounded $k_{6}$, all positive effective coefficients are constrained by ${\bar{k}}_{i}=\mathrm{softplus}\left(k_{i}\right)+\varepsilon_{k}$ for i∈{1,…,5,7,…,10}, with $\varepsilon_{k}=10^{-8}$. This mapping maintains positivity while preserving differentiability.

#### 2.2.2. Physical Polarization Decoupling Mechanism

Carrier transport in GaN HEMTs responds differently in the low-field conduction regime associated with $R_{\mathrm{on}}$ and the high-field saturation regime associated with $I_{\max}$ [16,17]. The model therefore forms a shared polarization base from the Al composition and an effective barrier thickness,

(4) $$t_{\mathrm{eff}}={\mathrm{softplus}}_{\beta }\left(t_{\mathrm{AlGaN}}-k_{6}\right)+\varepsilon_{t},\;\beta =500\;\mu m^{-1}$$

(5) $$P_{\mathrm{base}}=C\cdot x_{\mathrm{comp}}\cdot t_{\mathrm{eff}}$$

where C=100 µ$m^{-1}$ makes $P_{\mathrm{base}}$ dimensionless and $\varepsilon_{t}=10^{-6}$ µm prevents a zero effective thickness. The learned depletion depth $k_{6}$ is constrained to 2–8 nm through

(6) $$k_{6}=0.002+0.006\cdot \sigma \left({\mathrm{raw}}_{k_{6}}\right)$$

Separate positive exponents then map the shared polarization base to the low-field resistance and high-field current channels:

(7) $$P_{R_{\mathrm{on}}}=P_{\mathrm{base}}^{{\bar{k}}_{7}}$$

(8) $$P_{I_{\max}}=P_{\mathrm{base}}^{{\bar{k}}_{8}}$$

#### 2.2.3. Effective Steady-State DC Self-Heating Model

Here, “electrothermal” denotes the steady-state DC relation among a high-field power proxy, the endpoint maximum lattice temperature, and changes in the sweep-derived electrical indicators; time-dependent thermal dynamics are outside the present formulation. The model relates the high-field temperature rise to an effective DC thermal scale as

(9) $$T_{\max}=T_{\mathrm{amb}}+P_{\mathrm{proxy}}R_{\mathrm{th},\mathrm{macro}}$$

(10) $$P_{\mathrm{proxy}}^{\left(m\right)}=V_{\mathrm{ds},\max}I^{\left(m\right)}$$

(11) $$R_{\mathrm{th},\mathrm{macro}}=\frac{{\bar{k}}_{9}}{L_{\mathrm{total}}}$$

Here, $V_{\mathrm{ds},\max}=15$ V is the upper sweep limit, *I* is expressed in A/mm, and $P_{\mathrm{proxy}}$ is expressed in W/mm. Because $I_{\max}$ can occur below this limit, $P_{\mathrm{proxy}}$ is a calibrated high-field proxy rather than the exact pointwise dissipated power; $R_{\mathrm{th},\mathrm{macro}}$ is therefore an effective steady-state DC scale with units of K·mm/W [29]. With the lengths in (11) expressed in µm, ${\bar{k}}_{9}$ has units of K·mm·μm/W and controls how the power proxy is converted into the predicted $T_{\max}$. The ambient temperature is $T_{\mathrm{amb}}=300$ K, and $T_{\max}$ denotes the maximum lattice temperature at the sweep endpoint rather than a spatially resolved field or a switching-cycle temperature [30]. Equation (9) defines the baseline relation; its residual-enhanced iterative form is

(12) $$T_{\max}^{\left(m+1\right)}=T_{\mathrm{amb}}+R_{\mathrm{th},\mathrm{macro}}P_{\mathrm{proxy}}^{\left(m\right)}\left(1+\alpha_{T}n_{T}\right)+b_{T}$$

The resulting high-field temperature controls a bounded steady-state self-heating severity descriptor, which updates the current and provides a cross-regime modulation of the resistance prediction as

(13) $$I^{\left(m+1\right)}=I_{0}\left(1-\eta_{T}^{\left(m+1\right)}\right)$$

(14) $$R_{\mathrm{eff}}^{\left(m+1\right)}=R_{0}\left(1+\eta_{T}^{\left(m+1\right)}\right)$$

(15) $$\eta_{T}^{\left(m+1\right)}=0.8\tanh\left(\frac{{\bar{k}}_{10}{\left[T_{\max}^{\left(m+1\right)}-T_{\mathrm{amb}}\right]}_{+}}{0.8}\right).$$

where ${\left[z\right]}_{+}=\max\left\{z,0\right\}$ and ${\bar{k}}_{10}$ is a positive, time-independent self-heating sensitivity coefficient with units of K^−1^. It maps the predicted temperature rise to the dimensionless factor $\eta_{T}$; through (13) and (14), a larger $\eta_{T}$ reduces the high-field current and increases the effective resistance. Here, $R_{\mathrm{eff}}$ maps to low-bias $R_{\mathrm{on}}$, while $\eta_{T}$ is a cross-regime descriptor rather than the thermal state at the 0.4 V extraction point. Equations (12)–(15) form an *M*-step latent update initialized by $I^{\left(0\right)}=I_{0}$. After the final current update, $T_{\max}$ and $R_{\mathrm{eff}}$ are recomputed from the same descriptor to close the sweep-level prediction. Table 2 supports M=4 without using target labels to select the update depth. Closure is monitored by $|I_{0}\left(1-\eta_{T}\right)-I|/(|I|+\varepsilon_{c})$, where $\varepsilon_{c}=10^{-6}$ A/mm.

#### 2.2.4. Bounded Multiplicative Residual Correction

An unconstrained additive correction can replace rather than refine a physical baseline. In its generic form, such a correction is

(16) $$Y=Y_{\mathrm{phys}}+f\left(X\right)$$

where f(X) is an MLP correction. ET-DM-PGNN instead uses three target-specific MLPs and bounded multiplicative gates, so the learned terms remain proportional corrections around the gray-box bases [31]:

(17) $$\begin{array}{cc} n_{j}\left(X\right) & =c_{j}\tanh\left(f_{j}\left(X\right)/c_{j}\right),\;j\in \left\{I,R,T\right\},\\ I_{0} & =I_{\mathrm{cold}}\left(1+\alpha_{I}n_{I}\right)+b_{I},\\ R_{0} & =R_{\mathrm{cold}}\left(1+\alpha_{R}n_{R}\right)+b_{R}. \end{array}$$

The $R_{\mathrm{on}}$ and $T_{\max}$ residual branches use 5-128-128-1 MLPs with SiLU and hyperbolic-tangent activations, respectively, whereas the $I_{\max}$ branch uses a 5-64-64-1 MLP with hyperbolic-tangent activations. Each branch receives inputs min–max scaled to [−1, 1] using bounds fitted only to the corresponding training subset. The fixed correction budgets $\left[\alpha_{I},\alpha_{R},\alpha_{T}\right]=\left[0.4,0.2,0.1\right]$ and soft limits $\left[c_{I},c_{R},c_{T}\right]=\left[2,4,4\right]$ bound the three multiplicative factors to [0.2, 1.8], [0.2, 1.8], and [0.6, 1.4], respectively. These ranges retain an approximately linear response near zero while preventing unrestricted residual amplification. The additive offsets $b_{I}$, $b_{R}$, and $b_{T}$ are parameterized by hyperbolic tangents and limited to 20% of the corresponding training-target spans. The budgets were fixed before the external and level-held-out evaluations. The corrected electrical bases $I_{0}$ and $R_{0}$ and the bounded thermal residual $n_{T}$ enter the latent steady-state self-heating update in (12)–(15), rather than being appended to its output.

### 2.3. Physical Prior Initialization and Constrained Training

The positive effective coefficients are initialized as $\left[{\bar{k}}_{1},{\bar{k}}_{2},{\bar{k}}_{3},{\bar{k}}_{4},{\bar{k}}_{5},k_{6},{\bar{k}}_{7},{\bar{k}}_{8},{\bar{k}}_{9},{\bar{k}}_{10}\right]=\left[0.45,0.05,2.0,0.5,1.0,0.005,1.2,0.8,100,5\times 10^{-4}\right]$, with units inherited from (1)–(15). These scale-aware starting values place the cold electrical and thermal outputs in the same order of magnitude as the training targets and avoid saturated gradients at the first update; they are fixed before interstitial, external, and held-out evaluation. The three residual networks use the default PyTorch 2.10.0 linear-layer initialization, and the bounded output offsets start from zero. Optimization stability is assessed using positivity mappings, bounded residuals, scheduled learning-rate decay, convergence traces, and the three-seed directional and numerical-closure audits.

**Figure 2:**
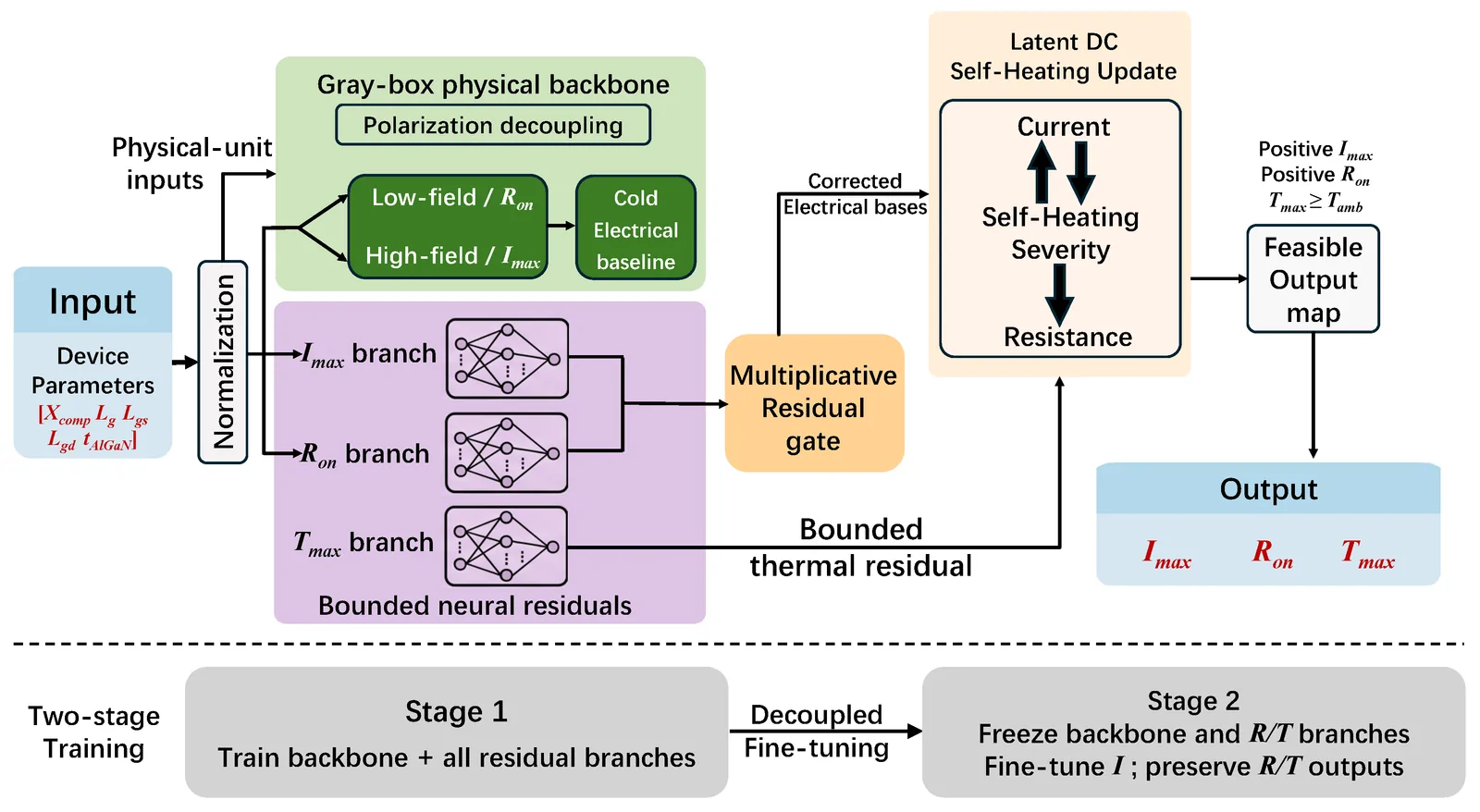
Workflow of ET-DM-PGNN. The gray-box electrical bases and bounded target-specific residuals are fused before a four-step unrolled steady-state DC self-heating update. Stage 2 fine-tunes the current branch while preserving the Stage-1 resistance and temperature responses.

The complete forward path is shown in Figure 2. Before target scaling and loss evaluation, the raw model outputs are mapped to the feasible physical domain:

(18) $$\begin{array}{cc} I_{\max} & ={\mathrm{softplus}}_{50}\left(I^{\left(M\right)}\right)+\varepsilon_{I},\\ R_{\mathrm{on}} & ={\mathrm{softplus}}_{50}\left(R_{\mathrm{eff}}^{\left(M\right)}\right)+\varepsilon_{R,o},\\ T_{\max} & =T_{\mathrm{amb}}+{\mathrm{softplus}}_{5}\left(T_{\max}^{\left(M\right)}-T_{\mathrm{amb}}\right). \end{array}$$

Here, ${\mathrm{softplus}}_{\beta }\left(z\right)=\beta^{-1}\ln\left(1+\exp\left(\beta z\right)\right)$, with β expressed in reciprocal units of *z*, and $\varepsilon_{I}$ and $\varepsilon_{R,o}$ are output floors of $10^{-8}$ in their respective output units. The selected slopes make the mappings nearly identity functions over the positive training range while retaining smooth feasibility at the boundary. Equation (18) enforces $I_{\max}>0$, $R_{\mathrm{on}}>0$, and $T_{\max}\geq T_{\mathrm{amb}}$ by construction. The physical outputs are subsequently scaled to [−1, 1] for supervised optimization; a tilde denotes a scaled target below. Stage 1 jointly optimizes the gray-box coefficients and all residual branches using Smooth L1 losses with transition parameter 0.01 for $I_{\max}$ and $T_{\max}$, an MSE loss for $R_{\mathrm{on}}$, and two auxiliary regularizers:

(19) $$\begin{array}{cc} L_{\mathrm{total}} & =L_{\mathrm{SmoothL}1}\left({\tilde{I}}_{\max}\right)+L_{\mathrm{MSE}}\left({\tilde{R}}_{\mathrm{on}}\right)\\ \; & \;+L_{\mathrm{SmoothL}1}\left({\tilde{T}}_{\max}\right)+\lambda L_{\mathrm{range}}+\gamma L_{\mathrm{smooth}}, \end{array}$$

The first regularizer is

(20) $$L_{\mathrm{range}}=E\;\left[\mathrm{ReLU}(I_{\mathrm{pred}}-2.0)\right]+E\;\left[\mathrm{ReLU}(R_{\mathrm{pred}}-50.0)\right].$$

Here, λ=0.1, and $I_{\mathrm{pred}}$ and $R_{\mathrm{pred}}$ in (20) denote predictions restored to physical units; the two upper thresholds are 2.0 A/mm and 50 Ω·mm, respectively. The range term discourages excessive current and resistance predictions and improves numerical conditioning. The temperature lower bound is already enforced structurally by (18) and is therefore not duplicated in $L_{\mathrm{range}}$. Because the factorial design contains only a few levels along each input dimension, the curvature of the bounded residual channels is also regularized:

(21) $$\begin{array}{cc} L_{\mathrm{smooth}} & =\frac{1}{5}\sum_{d=1}^{5}{∥\Delta_{d}n\left(X\right)/c∥}_{2}^{2},\\ \Delta_{d}n\left(X\right) & =n\left(X+he_{d}\right)-2n\left(X\right)+n\left(X-he_{d}\right). \end{array}$$

Here, $e_{d}$ is the unit vector of normalized input dimension *d*, h=0.2 is 10% of the normalized interval [−1,1], and division by c=[2,4,4] balances the residual channels. The weight γ=0.01 keeps this term subordinate to the supervised losses. These settings are fixed before evaluation; $L_{\mathrm{smooth}}$ regularizes the learned correction and is not a governing-equation residual.

**Figure 3:**
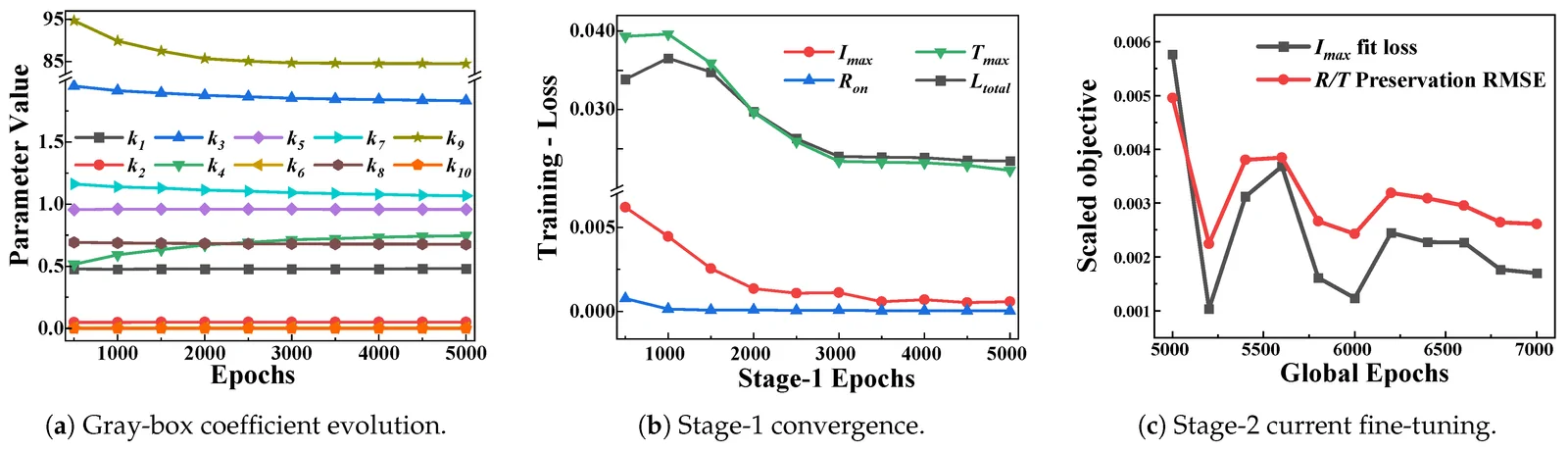
Optimization behavior of the full-budget ET-DM-PGNN model.

The two-stage schedule separates global latent DC self-heating calibration from target-specific refinement. Mini-batches contain 16 samples. In Stage 1, all gray-box coefficients and residual branches are optimized for 5000 epochs using Adam with an initial learning rate of $1\times 10^{-3}$ and weight decay of $1\times 10^{-4}$; the learning rate is halved every 1000 epochs. In Stage 2, the physical coefficients and the $R_{\mathrm{on}}$ and $T_{\max}$ branches are frozen, and the current branch is fine-tuned for 2000 epochs. The epoch budgets were retained after the logged full-budget losses reached a stable plateau without late-epoch divergence; Figure 3b,c reports the corresponding convergence traces. Because the current branch remains linked to the self-heating descriptor through (12)–(15), its objective includes a preservation term referenced to the frozen Stage-1 predictions:

(22) $$\begin{array}{cc} L_{S2} & =L_{\mathrm{SmoothL}1}\left({\tilde{I}}_{\max}\right)+\lambda L_{\mathrm{range}}\\ \; & \;+\lambda_{p}L_{\mathrm{MSE}}\;\left(\left[{\tilde{R}}_{\mathrm{on}},{\tilde{T}}_{\max}\right],{\left[{\tilde{R}}_{\mathrm{on}},{\tilde{T}}_{\max}\right]}_{S1}\right)\\ \; & \;+\gamma L_{\mathrm{smooth}}, \end{array}\;\lambda_{p}=1.$$

Stage 2 uses Adam with a learning rate of $5\times 10^{-4}$ and weight decay of $1\times 10^{-3}$; the same λ=0.1 and γ=0.01 are retained, and $\lambda_{p}=1$. The preservation term is evaluated in the scaled target space to balance resistance and temperature, limiting their drift while the high-field current correction is refined.

## 3. Numerical Results and Analysis

**Figure 4:**
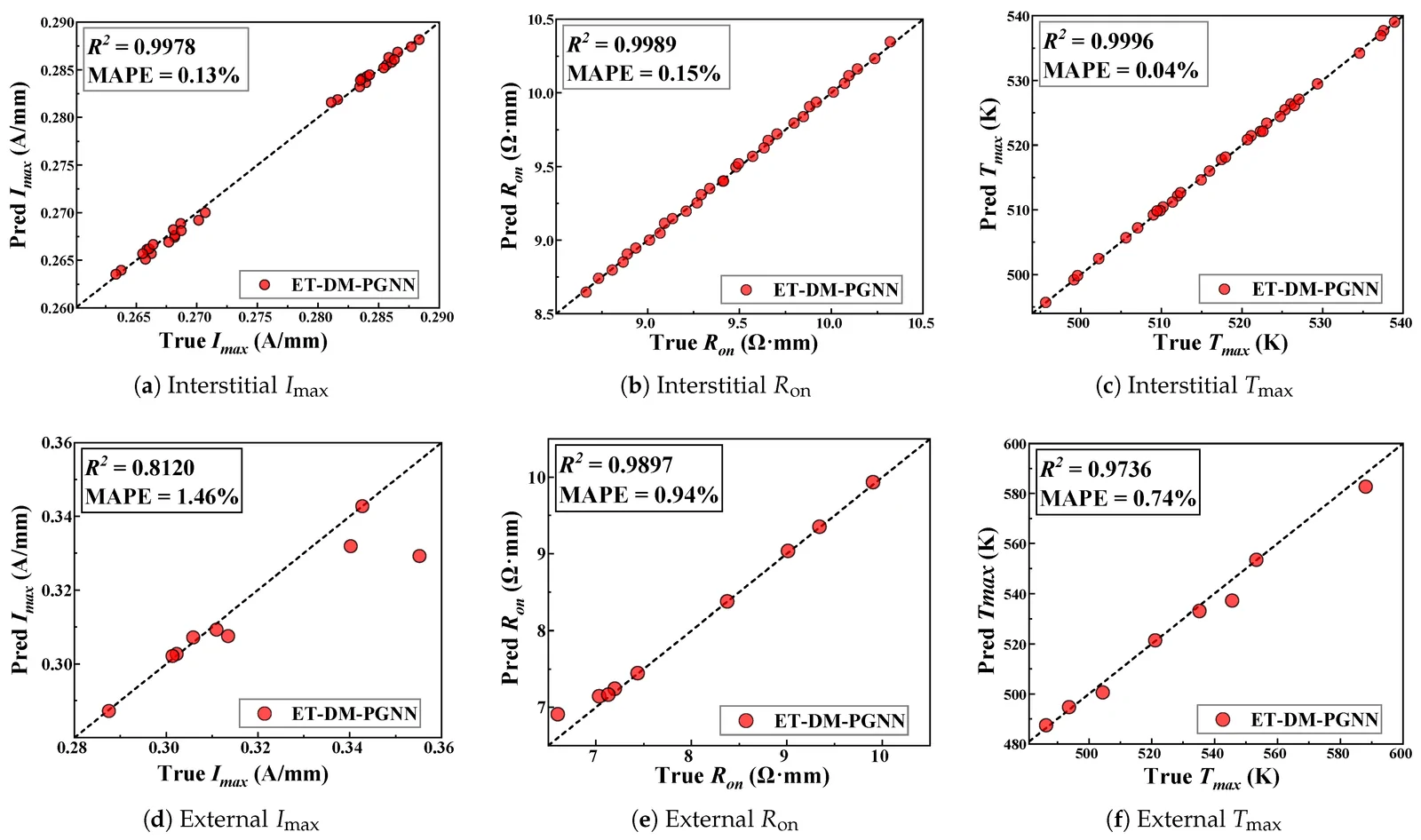
Parity plots for the full-budget ET-DM-PGNN model on the interstitial and external extrapolation sets. External cases follow the E1–E9 definitions in Table 4.

The numerical evaluation covers optimization behavior, closure of the four-step latent update, predictive accuracy, and the contribution of the main model components. Accuracy is tested on interstitial samples, external cases, and directional level-held-out splits. Figure 3 and Figure 4 and Table 3 use the 5000 + 2000-epoch schedule described in Section 2. The level-held-out audit uses a 200 + 80-epoch schedule with three random seeds per split; the Stage-1 learning-rate decay interval is reduced proportionally to 40 epochs, and all preprocessing parameters are fitted only to the training subset. Full-budget baseline comparisons and controlled component diagnostics are reported separately so that results obtained with different training budgets are not mixed.

### 3.1. Training Convergence and Numerical Closure

Before predictive accuracy is assessed, Figure 3 shows the two-stage optimization behavior. The embedded gray-box coefficients converge to finite effective values during Stage 1 (Figure 3a); in particular, $k_{6}$ remains within its prescribed depletion-depth interval, while ${\bar{k}}_{9}$ and ${\bar{k}}_{10}$ converge as the effective DC thermal scale and self-heating sensitivity coefficient, respectively. The Stage-1 objective decreases to a stable late-epoch plateau, and the bounded offsets remain within 20% of their target spans (Figure 3b). During Stage 2, only the current branch is updated, while the preservation term in (22) constrains changes in resistance and temperature (Figure 3c). The full-budget run uses the integer random seed 20260820; the directional and closure audits additionally use integer random seeds 20260821 and 20260822. All audited runs remain finite and satisfy the imposed output constraints.

The adequacy of the update depth is then examined independently of prediction error. After training, the same model is evaluated on all 365 available geometries (324 factorial-grid samples, 32 interstitial samples, and 9 external cases) over three random seeds. Table 2 reports the worst output difference and closure residual relative to an otherwise identical 12-step numerical reference; target labels are not used in this comparison. Four steps reduce the maximum output difference to 0.007573% and the maximum residual to $8.706\times 10^{-5}$, whereas six and eight steps produce only marginal additional changes. Changing the initial current from $I_{0}$ to $0.5I_{0}$ or $1.5I_{0}$ changes the four-step outputs by at most 0.0287%, with a maximum residual of $4.06\times 10^{-4}$. The four-step setting therefore provides a numerically closed and computationally compact update for the evaluated geometries.

### 3.2. Predictive Accuracy and Directional Validation

Predictive validation proceeds in increasing order of extrapolation difficulty. The 32 interstitial samples in Table 5 test interpolation between the structural levels of the 324-point factorial grid. The nine external cases in Table 4 probe structural settings beyond that grid through single-coordinate and coupled multicoordinate perturbations, including short channels, ultrathin barriers, high Al compositions, and long drain-side access regions. Finally, the directional level-held-out protocol removes one complete boundary level of a structural coordinate before training and evaluates that unseen level without test-based model selection.

**Table 4:** Nine external extrapolation cases in the order $\left[x_{\mathrm{comp}},L_{g},L_{\mathrm{gs}},L_{\mathrm{gd}},t_{\mathrm{AlGaN}}\right]$.

ID	Structural Parameters	Evaluated Regime
E1	[0.3, 0.12, 0.45, 1.45, 0.022]	Short channel and short source access
E2	[0.3, 0.1, 0.4, 1.45, 0.022]	Extreme short channel and source access
E3	[0.275, 0.12, 0.5, 1.45, 0.022]	Lower Al composition combined with a short channel
E4	[0.35, 0.2, 0.55, 1.55, 0.012]	High Al composition with an ultrathin barrier
E5	[0.37, 0.2, 0.55, 1.55, 0.010]	Extreme Al composition and barrier thickness
E6	[0.35, 0.2, 0.55, 1.55, 0.022]	High-composition polarization regime
E7	[0.3, 0.2, 0.5, 1.85, 0.022]	Extended drain-side access region
E8	[0.3, 0.2, 0.5, 2, 0.022]	Extreme drain-side access length
E9	[0.3, 0.15, 0.45, 1.85, 0.022]	Short channel combined with a long drain-side access region

**Table 5:** Ranges of the 32 interstitial test samples.

Variable	Range
$x_{\mathrm{comp}}$	[0.26, 0.28]
$t_{\mathrm{AlGaN}}$ (µm)	[0.018, 0.026]
$L_{\mathrm{gs}}$ (µm)	[0.53, 0.58]
$L_{\mathrm{gd}}$ (µm)	[1.5, 1.6]
$L_{g}$ (µm)	[0.175, 0.225]

The first two protocols are summarized by the parity plots in Figure 4. On the interstitial set (Figure 4a–c), all three targets achieve $R^{2}>0.997$, with MAPE values of 0.13%, 0.15%, and 0.04% for $I_{\max}$, $R_{\mathrm{on}}$, and $T_{\max}$, respectively, confirming high accuracy between the sampled grid levels. On the nine external cases (Figure 4d–f), the corresponding MAPE values remain at 1.46%, 0.94%, and 0.74%, while the $R^{2}$ values are 0.8120, 0.9897, and 0.9736. The lower current $R^{2}$ reflects the small external set and its narrow current range; the low MAPE indicates that the pointwise deviations remain limited. Cases E4 and E5 produce the most visible deviations. Both combine an above-training Al composition with a 12 or 10 nm barrier below the 15 nm minimum training level. In contrast, E6 retains the high Al composition and lateral geometry of E4 but uses a 22 nm barrier, with errors below 0.6% for all three targets. This contrast indicates that the coupled high-composition/ultrathin-barrier cases are more difficult under the present protocol than the composition shift alone. In (4)–(8), composition and effective thickness are compressed into the first-order proxy $P_{\mathrm{base}}=Cx_{\mathrm{comp}}t_{\mathrm{eff}}$. An increase in composition and a reduction in thickness can therefore partially compensate within this scalar proxy, although their coupled TCAD response need not remain equivalent near the ultrathin-barrier boundary. The comparison identifies this coupled extrapolation regime as difficult under the present data and training conditions without assigning a unique cause. E5, the most extreme coupled case, has errors of 7.19%, 4.75%, and 2.38% for $I_{\max}$, $R_{\mathrm{on}}$, and $T_{\max}$, respectively.

The third protocol isolates structural extrapolation along one coordinate at a time and explicitly controls preprocessing leakage. As reported in Table 6, the high-$x_{\mathrm{comp}}$ fold contains 243 training and 81 test rows, whereas each remaining fold contains 216 training and 108 test rows. The five folds reuse rows from the same 324-point factorial dataset; consequently, the 513 test-row occurrences are summarized by direction and are not treated as independent devices. Three-seed repetition quantifies optimization variability, and prediction averaging is performed only within each predefined fold.

Table 6 shows coordinate-dependent extrapolation behavior. The composition and three lateral-geometry folds yield errors of 0.439–6.403%; the largest is the $R_{\mathrm{on}}$ error for short $L_{\mathrm{gs}}$, consistent with the influence of source-side access geometry in (1)–(3). Holding out the complete 0.015 µm barrier level produces errors of 12.413–21.214%, identifying the ultrathin-barrier direction as the least reliable regime under the tested split and training budget. This experiment does not isolate whether the degradation originates from the polarization approximation, sparse thin-barrier support, or optimization duration. The folds are therefore interpreted as directional stress tests rather than an input-importance ranking; all outputs satisfy the imposed physical-domain constraints, and no architecture parameter is selected using held-out targets.

### 3.3. Baseline Comparison and Model Transferability

The final comparison evaluates whether the proposed combination of a gray-box backbone and bounded multiplicative residuals improves the overall three-target prediction profile. Table 3 compares ET-DM-PGNN with a purely data-driven MLP and an additive PGNN using the same TCAD data, normalization protocol, and full training budget. The MLP uses a shared 5-128-128-128-3 SiLU architecture with 34,179 trainable parameters. The additive PGNN retains the gray-box and target-specific branch architecture of ET-DM-PGNN, which has 39,440 trainable parameters, but replaces proportional residual gates with bounded additive corrections. This comparison is conducted on the interstitial and nine external cases; the baselines are not included in the shorter-budget level-held-out audit.

ET-DM-PGNN provides the most balanced accuracy across the three jointly predicted sweep-derived targets. The MLP has higher errors on both evaluation sets. The additive PGNN attains lower $I_{\max}$ MAPE because the current occupies a comparatively narrow range and can be corrected effectively by a small absolute offset. The same absolute correction is poorly matched to the broader, geometry-dependent scale of $R_{\mathrm{on}}$: it does not preserve a consistent fractional change as the cold resistance varies, which leads to 3.71% interstitial and 14.24% external MAPE. The multiplicative gate instead scales the correction with the physical resistance baseline and reduces these errors to 0.15% and 0.94%. Although the additive PGNN has a higher average $R^{2}$ on the external set than on the interstitial set, this does not indicate better extrapolation. $R^{2}$ is normalized by the target variance; the nine external cases span a larger resistance/temperature range than the interstitial set, whereas their MAPE values directly reveal the degradation.

To separate the roles of the main components, Table 7 reports a controlled 200 + 80-epoch diagnostic using the integer random seed 20260820, identical initialization, and the same mini-batch order. This reduced-budget diagnostic is reported separately from the full-budget comparison in Table 3. The “constrained ${\bar{k}}_{7}={\bar{k}}_{8}$” variant replaces the two exponents in (7) and (8) with one common value, thereby removing the separate low-field $R_{\mathrm{on}}$ and high-field $I_{\max}$ polarization responses. The “additive residual” variant replaces the proportional gate with an equally bounded absolute correction, and the “single thermal pass” variant uses one update instead of four.

Enforcing ${\bar{k}}_{7}={\bar{k}}_{8}$ mainly degrades current extrapolation, indicating that the distinct exponents in (7) and (8) provide useful flexibility for the low- and high-field polarization responses. Replacing proportional residuals with absolute corrections strongly degrades resistance and temperature, consistent with Table 3. The single-pass variant is competitive for some targets but nearly doubles external $R_{\mathrm{on}}$ error relative to the complete reduced-budget model. The four-step update is therefore retained for numerical closure and explicit cross-target linkage. Together with the MLP baseline, these diagnostics distinguish the contributions of the physical backbone, polarization decoupling, multiplicative residual learning, and the latent DC update.

The evaluation protocols answer different questions: the full-budget tests measure pointwise prediction error, the level-held-out splits test unseen structural boundaries, and the closure audit examines the numerical behavior of the four-step update. Agreement with held-out TCAD data establishes fidelity to the controlled numerical reference. Once trained, the surrogate maps five geometry variables to $I_{\max}$, $R_{\mathrm{on}}$, and $T_{\max}$ without repeating the coupled ATLAS sweep and post-processing for every candidate. This supports screening within the validated geometry and bias domain, while transfer to fabricated devices requires process-calibrated TCAD or measurements. The trained parameters apply to the $V_{\mathrm{gs}}=3$ V, 0–15 V drain-sweep protocol and sampled device family; other operating conditions, structures, or targets require corresponding data and recalibration.

Extending the present geometry-to-DC formulation to capacitance or switching would require bias-dependent terminal charge, time dependence, trapping dynamics, parasitic parameters, and consistently generated multibias or transient data [32].

## 4. Conclusions

ET-DM-PGNN is a gray-box surrogate for three GaN HEMT indicators extracted from one steady-state DC drain-bias sweep. The model combines separate high- and low-field polarization mappings, a latent DC self-heating update, bounded target-specific multiplicative corrections, and branch-selective training. Its outputs summarize current capability, conduction loss, and self-heating severity for geometry screening. Feasible-output mappings impose the required electrical and thermal domains, and the closure audit shows that four updates are sufficient for the evaluated geometries.

The model achieves $R^{2}>0.997$ on 32 interstitial samples, with MAPE values of 0.13%, 0.15%, and 0.04% for $I_{\max}$, $R_{\mathrm{on}}$, and $T_{\max}$, respectively. On nine external cases, the corresponding errors are 1.46%, 0.94%, and 0.74%. Four directional level-held-out tests yield errors of 0.44–6.40%, whereas the held-out 0.015 µm barrier level yields 12.41–21.21%. This result defines a validation limit under the tested protocol, although its origin cannot be isolated by the present experiment. ET-DM-PGNN therefore supports fixed-protocol geometry screening within the evaluated domain, with selected candidates verified by TCAD or measurement. The present parameters are validated against Silvaco ATLAS data at $V_{\mathrm{gs}}=3$ V using DC sweeps extending to $V_{\mathrm{ds}}=15$ V; other processes, biases, or dynamic targets require corresponding data and recalibration.

## Figures and Tables

**Table 1:** Range of device structural parameters.

Variables	Range
$x_{\mathrm{comp}}$	[0.25, 0.275, 0.3, 0.33]
$t_{\mathrm{AlGaN}}$ (µm)	[0.015, 0.022, 0.030]
$L_{\mathrm{gs}}$ (µm)	[0.50, 0.55, 0.60]
$L_{\mathrm{gd}}$ (µm)	[1.45, 1.55, 1.65]
$L_{g}$ (µm)	[0.15, 0.20, 0.25]

**Table 2:** Post-training latent-update closure sensitivity.

Steps *M*	Max. Output Change	Max. Residual
1	2.285835%	$2.688\times 10^{-2}$
2	0.341045%	$3.906\times 10^{-3}$
4	0.007573%	$8.706\times 10^{-5}$
6	0.000173%	$1.939\times 10^{-6}$
8	0.000022%	$1.832\times 10^{-7}$

**Table 3:** Full-budget baseline comparison.

Model	Test Dataset	$I_{\max}$ MAPE	$R_{\mathrm{on}}$ MAPE	$T_{\max}$ MAPE	Average $R^{2}$
MLP	Interstitial	1.05%	0.94%	0.33%	0.9429
	External	3.20%	4.27%	1.44%	0.7832
Additive PGNN	Interstitial	0.03%	3.71%	0.25%	0.7467
	External	0.20%	14.24%	0.78%	0.8884
ET-DM-PGNN	Interstitial	0.13%	0.15%	0.04%	0.9987
	External	1.46%	0.94%	0.74%	0.9251

**Table 6:** Leakage-controlled directional level-held-out results (three-seed ensemble, 200 + 80 epochs).

Held-Out Direction	Held Level	Train	Test	$I_{\max}$ MAPE	$R_{\mathrm{on}}$ MAPE	$T_{\max}$ MAPE
High $x_{\mathrm{comp}}$	0.33	243	81	0.594%	0.439%	0.512%
Low $L_{g}$	0.15 µm	216	108	4.307%	3.012%	1.641%
Low $L_{\mathrm{gs}}$	0.50 µm	216	108	3.770%	6.403%	1.516%
High $L_{\mathrm{gd}}$	1.65 µm	216	108	3.242%	3.733%	1.578%
Low $t_{\mathrm{AlGaN}}$	0.015 µm	216	108	21.002%	21.214%	12.413%

**Table 7:** Controlled reduced-budget component diagnostic (MAPE, %).

Variant	Interstitial			External		
	$I_{\max}$	$R_{\mathrm{on}}$	$T_{\max}$	$I_{\max}$	$R_{\mathrm{on}}$	$T_{\max}$
Complete ET-DM-PGNN	0.799	0.156	0.057	3.611	1.016	2.443
Constrained ${\bar{k}}_{7}={\bar{k}}_{8}$	2.142	0.242	0.036	5.443	2.032	1.594
Additive residual	0.579	9.765	36.644	1.291	24.957	38.608
Single thermal pass	0.260	0.115	0.234	2.945	1.971	2.078

## Data Availability

The processed TCAD datasets, Silvaco ATLAS 5.32.1.R input deck, and Python 3.11 training and evaluation scripts will be deposited in a public repository upon acceptance in accordance with the journal’s data-sharing requirements. A review copy is available from the corresponding author upon reasonable request.
